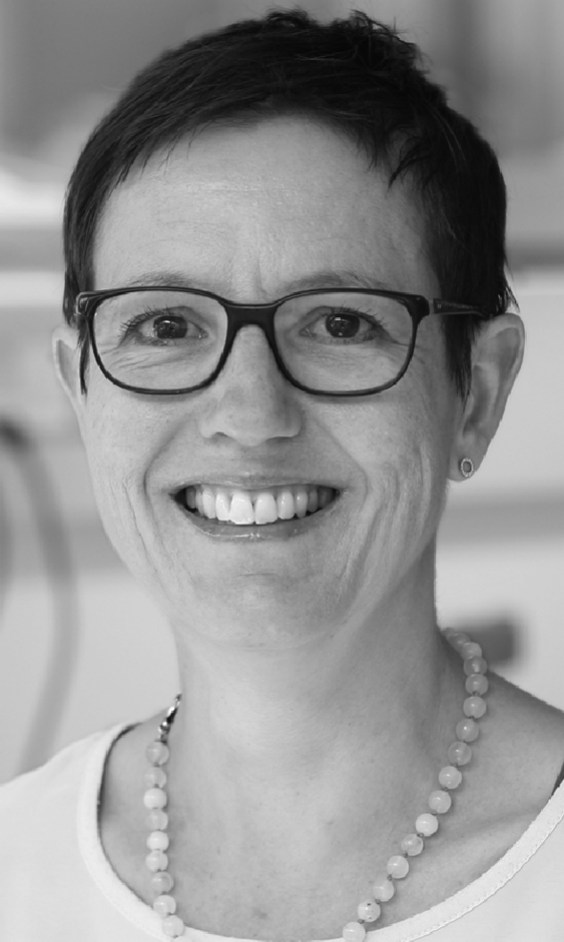# Cysteine‐Rich Antifungal Proteins from Filamentous Fungi are Promising Bioactive Natural Compounds in Anti‐*Candida* Therapy

**DOI:** 10.1002/ijch.201800168

**Published:** 2019-02-20

**Authors:** László Galgóczy, Annie Yap, Florentine Marx

**Affiliations:** ^1^ Institute of Plant Biology Biological Research Centre Hungarian Academy of Sciences Temesvári krt. 62 H-6726 Szeged Hungary; ^2^ Department of Microbiology Faculty of Science and Informatics University of Szeged Közép fasor 52 H-6726 Szeged Hungary; ^3^ Division of Molecular Biology Biocenter Medical University of Innsbruck Innrain 80–82 A-6020 Innsbruck Austria

**Keywords:** *Candida* spp., licensed antifungal drug, drug-resistance, antifungal protein, synthetic peptide

## Abstract

The emerging number of life‐threatening invasive fungal infections caused by drug‐resistant *Candida* strains urges the need for the development and application of fundamentally new and safe antifungal strategies in the clinical treatment. Recent studies demonstrated that the extracellular cysteine‐rich and cationic antifungal proteins (crAFPs) originating from filamentous fungi, and *de novo* designed synthetic peptide derivatives of these crAFPs provide a feasible basis for this approach. This mini‐review focuses on the global challenges of the anti‐*Canidia* therapy and on the crAFPs as potential drug candidates to overcome existing problems. The advantages and limitations in the use of crAFPs and peptide derivatives compared to those of conventional antifungal drugs will also be critically discussed.

## Graphical abstract

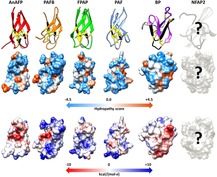

## 1 Introduction

Nowadays, the successful treatment of life‐threatening human fungal infections caused by *Candida* species has become more challenging as a consequence of (i) the current epidemiological changes in the genus, (ii) the limited number of effective antifungal drugs in the clinical therapy, and (iii) the emerging number of drug‐resistant isolates. These three factors indicate that recent therapies based on licensed antifungal agents have several limitations for a successful treatment of *Candida* infections and to overcome antifungal drug‐resistance of *Candida* species. Therefore, there is an urgent need for the development of new, alternative therapeutics and strategies in anti‐*Candida* therapy. Natural compounds such as antifungal peptides and proteins with potent anti‐yeast activity provide promising alternatives to the conventionally applied licensed antifungal drugs. In this review, we address the current global challenges in the treatment of *Candida* infections, and discuss the potential of secreted cationic, cysteine (Cys)‐rich antifungal proteins (crAFPs) originating from filamentous ascomycetes and the peptide derivatives thereof as promising candidates for future antifungal drug development.

## 2 Global Challenges in Treatment of *Candida* Infections

### 2.1 Epidemiological Changes

The genus *Candida* is represented by ∼150 yeast species, but only 15 of them have been described from human infections (candidiasis);^1^ and five species in particular (*Candida albicans*, *Candida glabrata*, *Candida parapsilosis*, *Candida tropicalis* or *Candida krusei*) have been identified from 95 % of all documented cases.^1^,^2^ *Candida* species belong to the normal human microbiota. In healthy individuals they colonize mainly the skin, mucosal surfaces of the oral cavity, gastrointestinal and urogenital tracts without causing any symptoms.^3^ Stress factors that weaken the immune system of the host may disturb this ecosystem and result in *Candida* overgrowth, causing non‐fatal, but disagreeable and very often recurrent superficial infections. Risk factors are for example surgical interventions, nosocomial bacterial infections, medications (antibiotics, hormone and chemotherapies), parenteral nutrition, mechanical ventilation as well as diseases that compromise the host‘s immune status (HIV infection, diabetes, hemato‐oncological malignancies).^4^, ^5^, ^6^, ^7^ Moreover, *Candida* pathogenicity is closely linked to the ability of the fungus to attach not only to the patient‘s tissues but also to synthetic medical devices (dentures, central venous catheters, shunts, etc.) where biofilm formation hampers therapeutic treatment efficacy.^5^ Depending on the overall health status of the patient, *Candida* cells may invade deeper into tissues and disseminate through the human body causing life‐threatening blood‐stream infections. *Candida* species are responsible for the most cases of invasive fungal infections with high mortality rates in immunocompromised patients all over the world.^8^ *C. albicans* is still the most frequently isolated *Candida* from human infections; however, the rapidly increasing number of non‐*albicans Candida* species (NAC) has been reported in the last years.^9^ NAC species show different distributions in diverse age groups and geographical locations. While *C. albicans* is the most prevalent pathogenic *Candida* species in patients aged up to 18 years, *C. parapsilosis* and *C. glabrata* is mostly isolated from neonates and elders, respectively. *C. glabrata* is the most common NAC species in North America and northern part of Europe, while *C. parapsilosis* in Southern Europe, Africa, India, and Latin America.^10^,^11^ In addition, *Candida auris* as an emerging human pathogenic yeast represents a serious global health threat nowadays.^12^ These recent epidemiological changes have an important clinical impact as the NAC species show diminished susceptibility to the first‐line therapeutically applied antifungal agents, which are generally effective against *C. albicans*.^13^

### 2.2 Limitations in Clinical Therapy

Today, only three groups of licensed antifungal drugs are applied for treatment of life‐threatening blood‐stream *Candida* infections (candidemia) and invasive candidiasis when the infection affects more than one organ of the patient. These are the triazoles (fluconazole, voriconazole, posaconazole), the echinocandins (caspofungin, micafungin, anidulafungin), and polyenes (different formulations of amphotericin B).^14^ Echinocandins and triazoles are better tolerated by the patients than amphotericin B. Due to the risk of high toxicity (nephrotoxicity), amphotericin B is no more considered as an option in the treatment. Lipid formulations of amphotericin B diminish the nephrotoxic effect, but are significantly more expensive than the well‐tolerated fluconazole and more toxic than echinocandins, hence its application is considered only in specific situations.^15^ As fluconazole is inexpensive and readily available worldwide, it is the most frequently prescribed triazole to treat *Candida* infections.^16^ However, voriconazole is more efficient than fluconazole, but considering the high therapeutic cost it is prescribed only when the infective *Candida* strain is fluconazole‐resistant.^17^ Posaconazole is approved for use as a prophylactic agent,^18^ but its therapeutic application is considered against fluconazole‐, voriconazole‐ and cross‐resistant *Candida* isolates.^19^ Recently, echinocandins are considered as the most effective antifungals and are used extensively to treat candidemia and invasive candidiasis due to the broad‐spectrum against *Candida* species and generally mild adverse effects, but their application is limited by the high costs of echinocandin therapy.^20^ Despite the successful introduction and application of the above mentioned three antifungal drug groups in the clinical therapy, *Candida* infections with fatal outcome are becoming more frequent as a consequence of emerging resistance mechanisms.^21^

### 2.3 Emergence of Antifungal Drug‐Resistance

All developed resistance mechanisms directly or indirectly have an impact on the antifungal drug target. Amphotericin B binds to ergosterol, the main sterol in the fungal cell membrane, and forms membrane‐spanning pores resulting in the lysis of the fungal cell. Alteration of the cytoplasmic membrane lipid composition reduces the affinity of amphotericin B for the cell membrane and renders the fungus less susceptible or even resistant against this antifungal agent.^22^ Azoles inhibit the biosynthesis of ergosterol, causing the intracellular accumulation of toxic sterol intermediates and inducing cell membrane stress. Overexpression of different efflux pumps and enzymes playing a role in ergosterol biosynthesis or their mutations in the drug‐binding domain contribute to the resistance mechanism.^22^ Echinocandins are noncompetitive inhibitors of 1,3‐β‐D‐glucan synthase, an integral component of the fungal cell wall biosynthesis, and its dysfunction leads to loss of cell wall integrity. The mutation in the catalytic domain of the (1,3)‐β‐D‐glucan synthase is the most common resistance mechanism against this drug class.^22^ As fluconazole and echinocandins are the first‐ or second‐line applied antifungals, several *Candida* species show resistance to these medications. Global surveillance programs demonstrated that the increasing incidence of fluconazole resistance is a particular problem with *Candida* infections, especially with *C. glabrata* and *C. krusei*. However, the resistance against echinocandins was detected only within two years after their clinical launch and almost 3 % of *C. glabrata* isolates have already developed resistance mechanisms.^21^,^23^,^24^ Multidrug resistance to azoles, echinocandins, and polyenes is still uncommon within the genus, but its emergence in several *Candida* species has been reported and points towards an increasing trend among *C. glabrata* and *C. auris* isolates.^25^

## 3 Anti‐*Candida* Proteins from Filamentous Ascomycetes

### 3.1 Origin and Phylogeny

Filamentous ascomycetes, especially the members of the class Eurotiomycetes are a rich source for extracellular crAFPs.^26^ They share common features such as a low molecular mass, the presence of six to eight Cys residues that form three to four intramolecular disulfide bonds which provide a high stability against protease degradation, extreme temperatures and within a broad pH range. Another common feature is their cationic character based on a high amount of arginine (Arg), lysine (Lys) and histidine (His) residues in the primary structure. The secreted crAFPs differ in their amino acid sequences, but conserved homologous parts in the primary structure can be identified at the flanking regions of Cys.^27^ A recently published phylogenetic analysis demonstrated that crAFPs can be divided into four different groups based on these conserved regions: proteins with characteristic amino acid motifs to (1) *Aspergillus giganteus* antifungal protein (AFP‐clade), (2) *Penicillium chrysogenum* antifungal protein (PAF‐clade), (3) *Penicillium brevicompactum* ‘bubble protein’ (BP‐clade), and (4) *Neosartorya* (*Aspergillus*) *fischeri* antifungal protein 2 (NFAP2‐clade).^26^ It has to be noted that one species could produce more than one AFP belonging to the same clade, *e. g*. PAF and PAFB (PAF‐clade proteins) from *P. chrysogenum*;^28^ or to different clades *e. g*. NFAP (PAF‐clade protein) and NFAP2 (NFAP2‐clade protein) from *N. fischeri*.^29^ The crAFPs are mainly effective against filamentous fungi, but potent anti‐yeast activity has been reported recently only for some representatives belonging to the PAF‐ (AnAFP, FPAP, PAF, PAFB), BP‐(BP), and NFAP2‐clade (NFAP2), respectively.^26^,^28^, ^29^, ^30^, ^31^, ^32^ These proteins, their origin and physicochemical properties are summarized in Table 1.

**Table 1:** Amino acid sequence and *in silico* predicted physicochemical properties of crAFPs with anti‐yeast activity.

Protein	Origin (isolate)	UniProtKB ID^33^	Number of amino acids	Molecular weight (kDa)^34^	Number of Cys	Number of Lys/Arg/His	Theoretical isoelectric point^34^	Estimated charge at pH 7^35^	GRAVY^34^
PAF‐clade									
LSKYGGECSLEHNTCTYRKDGKNHVVSCPSAANLRCKTDRHHCEYDDHHKTVDCQTPV									
AnAFP	*Aspergillus niger* KCTC 2025	A0A117E0B2	58	6.6	6	5/3/6	7.14	+1.2	−1.076
LEYWGKCTKAENRCKYKNDKGKDVLQNCPKFDNKKCTKDGNSCKWDSASKALTCY									
FPAP	*Fusarium polyphialidicum* SZMC 11042	E1UGX4	55	6.4	6	12/1/0	9.10	+5.7	−1.291
AKYTGKCTKSKNECKYKNDAGKDTFIKCPKFDNKKCTKDNNKCTVDTYNNAVDCD									
PAF	*Penicillium chrysogenum* Q176	Q01701	55	6.3	6	13/0/0	8.93	+4.7	−1.375
LSKFGGECSLKHNTCTYLKGGKNHVVNCGSAANKKCKSDRHHCEYDEHHKRVDCQTPV									
PAFB	*Penicillium chrysogenum* Q176	A0A167QQK7	58	6.5	6	8/2/6	8.83	+5.2	−1.031
BP‐clade									
DTCGSGYNVDQRRTNSGCKAGNGDRHFCGCDRTGVVECKGGKWTEVQDCGSSSCKGTSNGGATC									
BP	*Penicillium brevicompactum* Dierckx	G5DC88	64	6.6	8	4/4/1	7.70	+0.9	−0.867
NFAP2‐clade									
IATSPYYACNCPNNCKHKKGSGCKYHSGPSDKSKVISGKCEWQGGQLNCIAT									
NFAP2	*Neosartorya fischeri* NRRL181	A0A1D0CRT2	52	5.6	6	7/0/2	9.02	+5.2	−0.731

GRAVY: grand average of hydropathy value. Bold and underlined letters indicate the γ‐core motif(s).

### 3.2 Protein Structure

**Figure 1:**
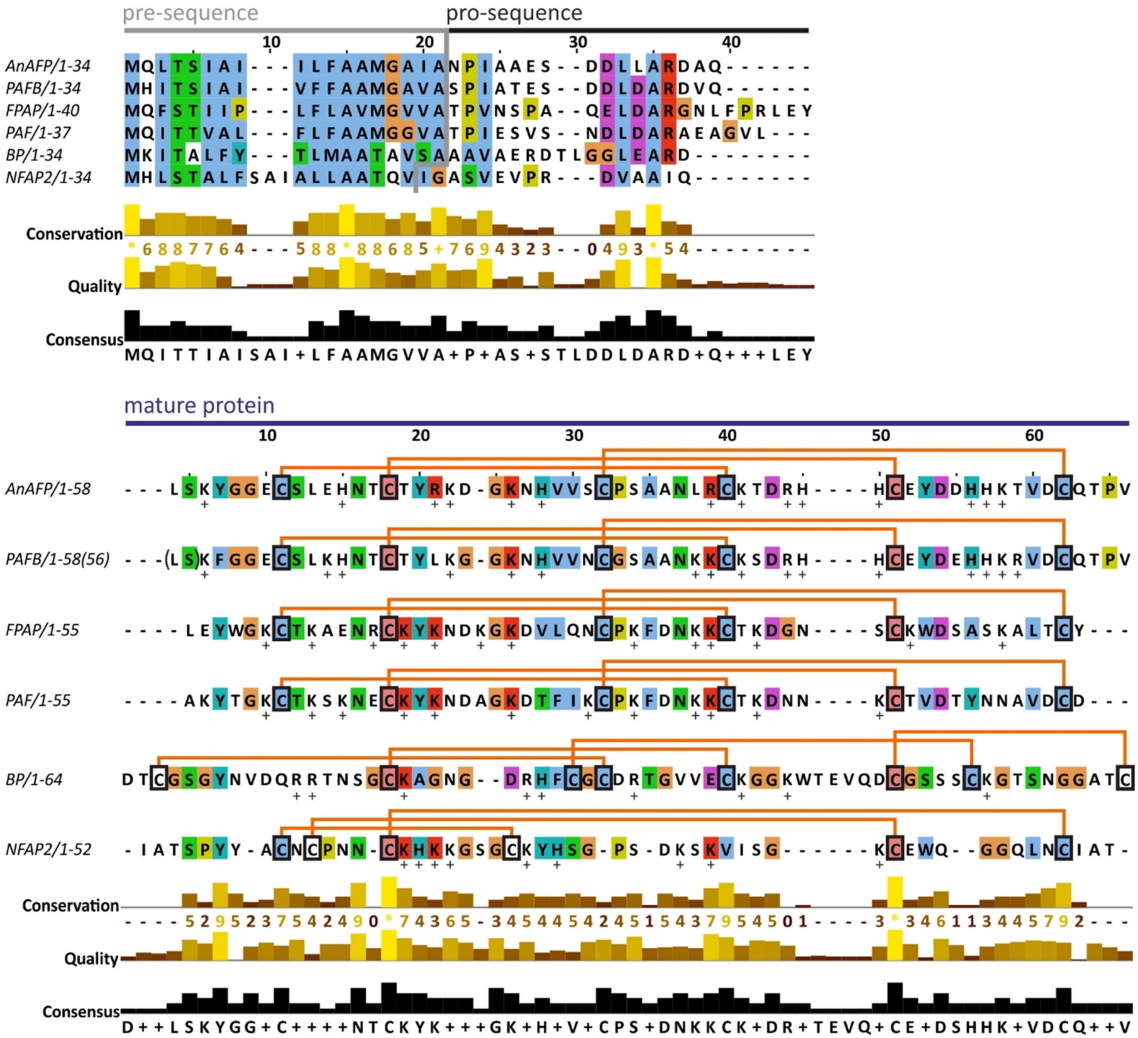
Primary structure of anti‐yeast crAFPs isolated from Eurotiomycetes. ClustalW multiple alignment of the prepro‐sequences and the sequences of mature proteins were generated with the BioEdit program^36^ and visualized with Jalview version 2.10.3b1.^37^ The cleavage site of the predicted signal sequence (pre‐sequence indicated by grey line) was predicted by SignalP1 4.1 server.^38^ Connective orange lines between Cys residues (C in black frame) indicate disulfide bridge formations. The ClustalX default color scheme was applied in the alignment (http://www.jalview.org/help/html/colourSchemes/clustal.html) to show similarity.^37^ Brackets in the sequence of PAFB indicate that this protein is expressed in 58 amino acid full‐length form into the supernatant, however the leucine (L) and the serine (S) are cleaved from the N‐terminus with time, generating the 56 amino acid sfPAF form which served for the tertiary structure determination.^28^

The crAFPs are expressed as prepro proteins and the pre‐ and pro‐sequence are cleaved off from the N‐terminus during the maturation process and secretion (Figure 1).^27^ The proper maturation of crAFPs is crucial for the full antifungal activity.^44^ The primary structure of crAFPs belonging to distinct clades show low homology, but it contains six or eight Cys residues. Three intramolecular disulfide bridges adopting a common *abcabc* pattern are formed in the members of the PAF‐ (AnAFP, PAF, PAFB, FPAP) and NFAP2‐clades (NFAP2); or four disulfide bonds are formed connecting the loop region to sheet 1, and the sheet 1 to the base of sheet 2, as it is found in the members of the BP‐clade (BP) (Figure 1).^26^,^45^ The correct disulfide bridge pattern is essential for the structural stability and the function.^44^

**Figure 2:**
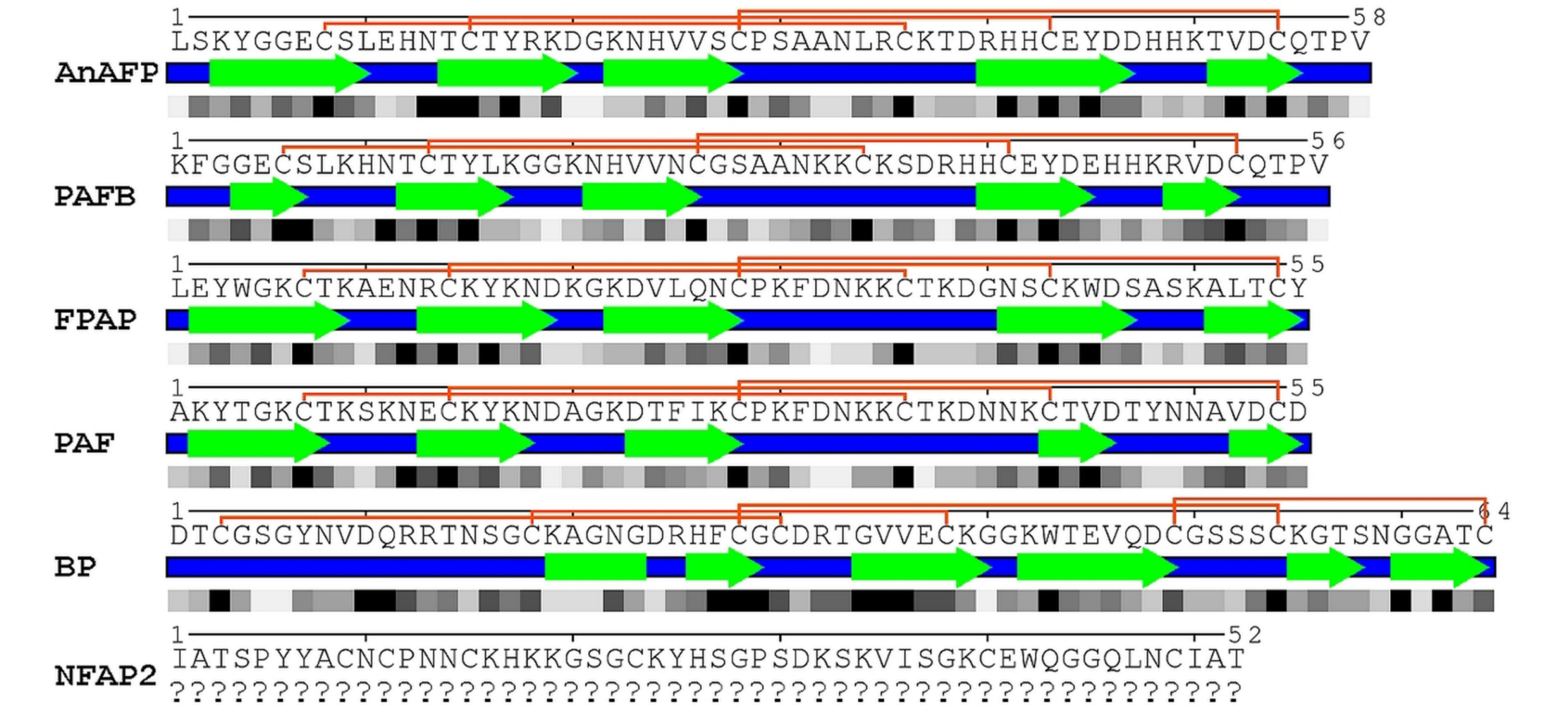
Secondary structure and relative solvent accessibility (RSA) of anti‐yeast crAFPs isolated from Eurotiomycetes. Linear representation of secondary structures and RSA were generated from the respective annotated (PAFB, PAF, BP) or predicted (AnAFP and FPAP) .pdb file of tertiary structure (Protein Data Bank IDs: PAFB‐2nc2, PAF‐2mhv, BP‐1uoy) with POLYVIEW‐2D server^39^ and revised based on the tertiary structure visualized with UCSF Chimera software.^40^ Blue lines and green arrows indicate the loop and β‐strand regions, respectively. Connective orange lines between Cys residues indicate disulfide bridge formations. Below the secondary structure, the RSA is indicated, in which the black and white squares represent completely buried (0‐9 RSA) and fully exposed (90‐100 RSA) amino acids, respectively. Putative tertiary structure of AnAFP and FPAP was predicted *in silico* by I‐Tasser,^[41]^ and refined by ModRefiner.^42^ Tertiary structure of PAF (Protein Data Bank ID: 2mhv) served as a template to model the structure of AnAFP and FPAP. Question marks indicate that the secondary structure of NFAP2 is under investigation.

Electronic circular dichroism spectroscopy of PAF, PAFB, NFAP2 and structural prediction of AnAFP and FPAP (in this review) revealed that these crAFPs have a common β‐pleated conformation due to the presence of numerous β‐strands in their secondary structure (Figure 2).^28^,^29^,^46^

**Figure 3:**
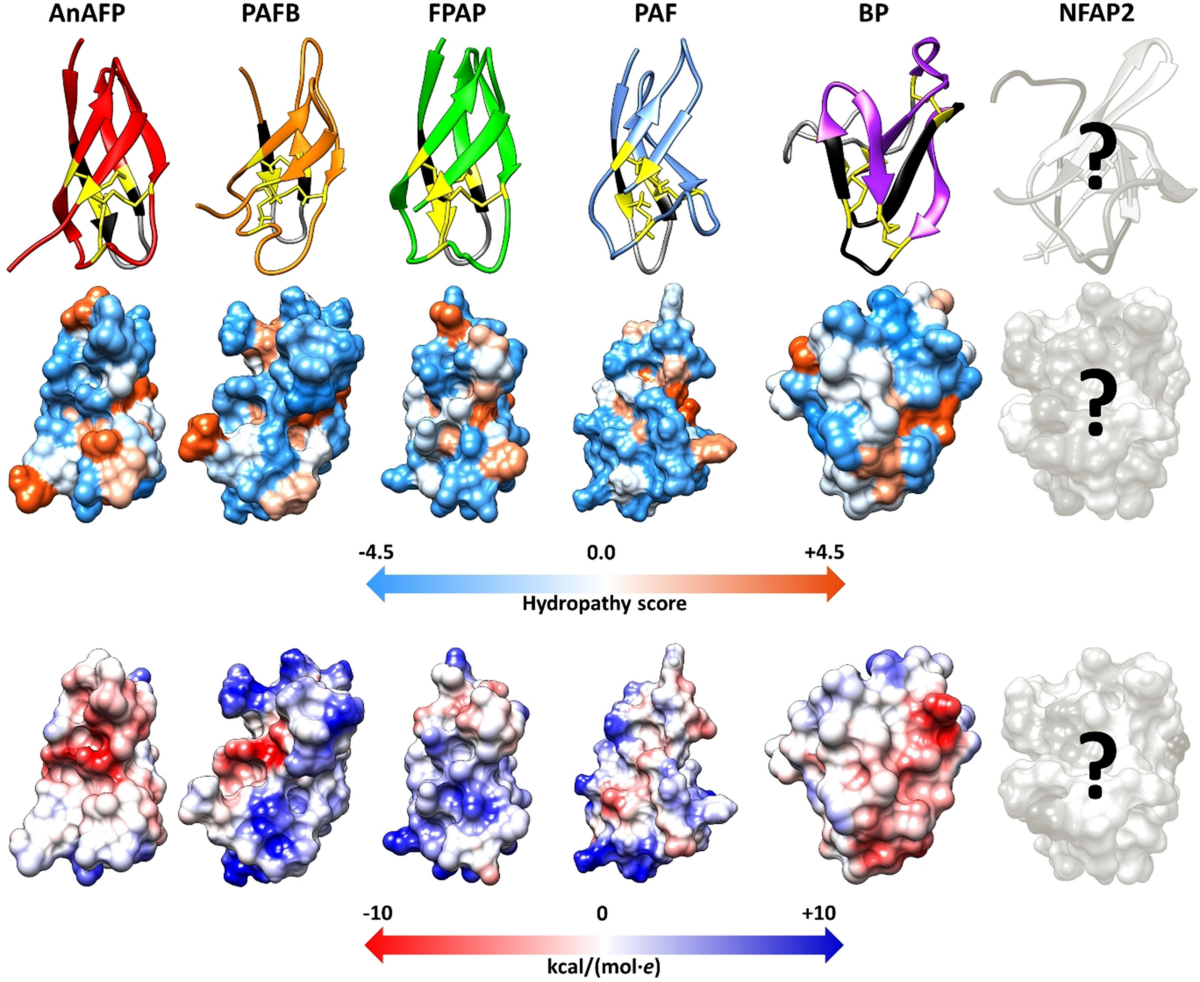
Tertiary structure, hydrophobicity (Kyte‐Doolittle scale of amino acids with colors ranging from blue for the most hydrophilic to white at 0.0 to orange for the most hydrophobic) and electrostatic surface (Coulombic surface coloring of amino acids with colors ranging from dark blue for positive charge to white at 0 to red for negative charge) (from up to down) of anti‐yeast crAFPs isolated from Eurotiomycetes. The first loop region and the γ‐core motif is indicated by black and grey, respectively. Cys residues and disulfide bridges are marked in yellow and yellow lines, respectively. All structures were generated with UCSF Chimera visualization software using the respective annotated (Protein Data Bank IDs: PAFB‐2nc2, PAF‐2mhv, BP‐1uoy) or predicted (AnAFP and FPAP) .pdf file of tertiary structure, respectively.^40^ Putative tertiary structure of AnAFP and FPAP was predicted *in silico* by I‐Tasser,^41^ and refined by ModRefiner.^42^ Based on the Ramachandran plot analysis,^43^ 96.4 % and 100 % of the residues are in the favored and allowed position in AnAFP and FPAP, respectively. Tertiary structure of PAF (Protein Data Bank ID: 2mhv) served as a template to model the structure of AnAFP and FPAP. Question marks indicate that the tertiary structure, hydrophobicity and electrostatic surface of NFAP2 are under investigation.

Based on nuclear magnetic resonance (NMR) spectroscopy,^28^,^47^ X‐ray crystallography,^45^ and *in silico* homology modeling prediction experiments (in this review) the members of PAF‐ and BP‐clade anti‐yeast proteins share a very similar overall tertiary structure; a β‐barrel topology, in which five antiparallel β‐strands connected by loops create two orthogonally packed β‐sheets. The loops are solvent exposed and flexible suggesting a role in the binding to the fungal target cell.^47^,^48^ The above discussed structural features have been confirmed experimentally for PAF,^47^,^49^ PAFB,^28^ and BP,^45^ and predicted for AnAFP, and FPAP (the last two in this review, Supporting Information) (Figure 3). NMR investigations of NFAP2 are in progress.^50^ These proteins have an amphipathic surface with alternating positively‐ and negatively‐charged patches (Figure 3). It is assumed that apart from the correct disulfide bonding between the Cys residues, a hidden central hydrophobic core coordinates the proper folding and the formation of a stable protein structure.^44^,^47^

### 3.3 Recombinant Production

From economic view, one of the limiting factors for future application of crAFPs as anti‐*Candida* compounds is the low‐yield production by the native producers.^27^ To meet this requirement, a *P. chrysogenum*‐based expression system was developed for the optimal bulk production of PAF,^46^ PAFB,^28^ and NFAP2.^50^ This system is reliable considering the correct protein processing, folding, and disulfide bond formation.^46^,^50^ Applying *P. chrysogenum* as expression host, crAFPs do not undergo any posttranslational modifications, except for the required cleavage of the prepro‐sequence (Figure 1) and folding. This recently reported system represents the perfect tool for cost‐effective generation of crAFPs in high yields.^46^,^50^ Notably, *P. chrysogenum* is fermentable and generally recognized as safe (GRAS) organism by the US Food and Drug Administration.^51^

### 3.4 Chemical Synthesis

In comparison with time‐intensive protein production by fermentation, chemical synthesis of full‐length crAFPs is fast, but still not cost‐effective considering the final protein yield. Different *de novo* designed crAFP variants were chemically synthesized in low amounts for preliminary structural investigations and antifungal activity testing before the more time‐intensive generation of the respective genetically engineered fungal strains was started to produce these antifungal proteins in high amounts (unpublished). Studies regarding the synthetic production of PAF and NFAP2 proved that the method of native chemical ligation of peptide fragments using solid‐phase fluorenylmethyloxycarbonyl (Fmoc) chemistry combined with an additional oxidation step represents a fast and reliable method to synthesize functionally active crAFPs.^50^,^52^ Moreover, the specific protection of Cys sulfhydryl groups within oxidative conditions allows the formation of the correct disulfide bridge pattern or their variation to investigate the structure‐function relation in crAFPs.^50^,^52^ The current limitation of this method is the risk for disulfide bond formation deviating from the correct pattern, and for bond disruption in the presence of reducing agents during the synthesis process; however several solutions are available to overcome these methodological problems.^52^,^53^ Furthermore, this method is valuable to modify the amino acid sequence to discriminate essential from non‐essential structural elements determining crAFPs function. Considering the fact that Fmoc solid‐phase peptide synthesis is becoming more economic nowadays,^54^ this chemical method could provide an alternative for the industrial‐scale production of ultrapure crAFPs in the future.

## 4 Anti‐*Candida* Activity of crAFPs

### 4.1 *In Vitro* Anti‐*Candida* Activity

The *in vitro* susceptibility of different clinically relevant *Candida* species to crAFPs is summarized in Table 2. However, it has to be noted that the detected minimal inhibitory concentrations (MICs) strongly depend on the applied test medium. It was demonstrated that the MIC of NFAP2 is higher in a standard high cationic clinical susceptibility (HCCS) test medium mimicking the composition of human extracellular environment than in a low ionic strength medium optimized for the investigation of crAFP activity (Table 2).^50^ Generally, it is typical for all members of this protein group that high ionic strength media decrease the *in vitro* antifungal efficacy.^27^,^44^ In contrast to NFAP2,^29^ all anti‐yeast crAFPs tested so far inhibit the growth of filamentous fungi,^27^,^28^,^32^ but were found inactive against yeasts in standard HCCS test medium, for example, the *P. chrysogenum* antifungal proteins PAF and PAFB show very low activity against different *Candida* species in RPMI 1640 (unpublished results). One possible explanation for this phenomenon is provided by the mechanism of action of antifungal plant defensins. These cationic antifungal proteins electrostatically attach to the negatively charged phospholipid heads in the fungal plasma membrane before they exert their antifungal effect. This electrostatic interaction is cation sensitive by competition between the cationic antifungal proteins and cations present in the medium for the binding sites.^55^ Antifungal efficacy of BP on *Candida* species has not been tested yet, but the reported strong inhibitory activity on baker‘s yeast *Saccharomyces cerevisiae* suggests a high efficacy to inhibit the growth of other yeasts.^31^

**Table 2:** Minimal inhibitory concentrations (MICs) of crAFPs on different *Candida* species and their predicted binding affinity to human serum albumin (HSA).

Species	MIC (μM)					
	AnAFP	PAFB^*^	FPAP	PAF^*^	NFAP2	
	Applied medium					
	standard^30^	diluted^28^	standard^32^	diluted^28^	standard^50^	diluted^50^
*C. albicans*	8–15	1	>24	4	36	1
*C. glabrata*	n.d.	0.6	>24	2.5	2	0.3
*C. guilliermondii*	n.d.	n.d.	>24	n.d.	0.6	0.3
*C. inconspicua*	n.d.	n.d.	24	n.d.	n.d.	n.d.
*C. krusei*	n.d.	0.6	>24	5	72	2
*C. lipolytica*	n.d.	n.d.	24	n.d.	n.d.	n.d.
*C. lusitaniae*	n.d.	n.d.	24	n.d.	>18	0.6
*C. norvegica*	n.d.	n.d.	24	n.d.	n.d.	n.d.
*C. parapsilosis*	n.d.	0.6	>24	2.5	18	0.3
*C. tropicalis*	n.d.	n.d.	>24	n.d.	>18	0.3
*C. zeylanoides*	n.d.	n.d.	>24	n.d.	n.d.	n.d.
**Predicted binding affinity to HSA** 65						
ΔG (kcal/mol)	−9.15	−8.88	−13.80	−11.09	−12.16	
Kd (M)	1.95e‐07	3.07e‐07	7.52e‐11	7.33e‐09	1.21e‐09	

*The MIC of PAF and PAFB on NAC was determined as described.^26^ n.d.: not determined. Media applied in susceptibility tests: AnAFP – Yeast Mold Medium (YM: 1 % glucose, 0.3 % malt extract, 0.5 % peptone, and 0.3 % yeast extract (*w/v*)), PAF and PAFB – Ten‐fold diluted Potato Dextrose Broth (PDB; Becton Dickinson), FPAP – Low Cationic Agar Medium (LCM: 2 % glucose, 0.1 % yeast extract, 0.05 % peptone (*w/v*)), NFAP2 – Roswell Park Memorial Institute 1640 medium (RPMI 1640; Sigma‐Aldrich) as standard medium, and diluted Low Ionic Strength Broth Medium (LCM: 0.5 % glucose, 0.25 % yeast extract, 0.0125 % peptone (*w/v*)). ΔG is the binding free energy, Kd is the dissociation constant. PPA‐Pred2 (Protein‐Protein Affinity Predictor) server for miscellaneous complexes was applied to calculate ΔG and Kd.^65^

### 4.2 Anti‐*Candida* Activity in Drug Combination

In combinatorial antimicrobial therapy two or more drugs are administered simultaneously to treat an infection. This treatment is applied when the infectious agent shows low susceptibility or even resistance to one drug and/or the prolonged high‐dosage monotherapy can cause severe side‐effects in the host. Combination of antifungal agents represents a more effective therapy than their single application by shortening the treatment period and decreasing the administered drug concentrations to avoid toxic adverse effects.^56^ Studies successfully demonstrated synergistic antifungal effects *in vitro* and *in vivo* when crAFPs and licensed antifungals or other drugs possessing a secondary antifungal effect were combined.^27^,^57^,^58^,^59^ As for anti‐*Candida* activity, the co‐administration of NFAP2 and fluconazole in standardized clinical microbiological susceptibility tests inhibited the *in vitro* growth of *C. albicans* and *C. parapsilosis* in a synergistic way, but was neutral against *C. krusei*.^50^ The applicability of crAFPs *in vivo* was further shown in a mouse infection model, where the synergistic anti‐*Candida* efficacy of NFAP2 in combination with fluconazole was proven recently.^60^ In contrast, no antagonistic mode of actions have been observed so far, which underlines the potential of crAFPs for anti‐*Candida* polytherapy.^27^,^50^

### 4.3 Anti‐*Candida* Mechanisms

The anti‐*Candida* mechanisms of crAFPs are not fully understood, and available data exist only for NFAP2,^29^,^50^ PAF,^26^,^28^ and PAFB.^28^ NFAP2 causes prompt plasma membrane disruption in *C. albicans* indicating a fast and fungicidal mechanism of action even in high cationic medium under clinical susceptibility test conditions.^29^,^50^ The fungicidal mechanism reduces the risk of resistance development, and further underlines the antifungal potential of NFAP2 under *in vivo* conditions. PAF and PAFB also act candidacidal, but in contrast to NFAP2, these proteins are not effective in high ionic strength media.^28^ Their mode of action was extensively investigated in diluted complete media.^26^,^28^ Both crAFPs are internalized by *C. albicans via* an energy‐dependent mechanism before the plasma membrane of the *Candida* cells is disrupted.^26^,^28^ Apart from protein uptake, the induction of reactive oxygen species is closely linked with the antifungal activity of PAF, which was shown to be also effective against *C. albicans* biofilm formation.^26^ This antifungal mode of action of PAF closely resembles that of PAF26, a *de novo* designed, highly cationic synthetic antifungal hexapeptide.^61^ The observations made with NFAP2, PAF and PAFB suggest that crAFPs of different crAFP groups interact with distinct target(s) involved in the anti‐*Candida* activity. Further detailed investigations are necessary to identify the interaction molecules.

## 5 Potential Therapeutic Applications and Limitations

Several studies unambiguously demonstrated the fungal selectivity of crAFPs as these proteins exhibit neither hemolytic nor cytotoxic effects on mammalian cells *in vitro*.^26^,^28^,^60^,^62^,^63^ Intranasal and topical application of PAF proved to be safe in a toxicity testing in mice: no adverse effects were detected when the protein was administered at its highest *in vitro* inhibitory concentration for *Aspergillus fumigatus*.^59^ In a subsequent study the *in vivo* antifungal potency of intraperitoneally administered PAF was demonstrated with a murine pulmonary aspergillosis model. The intranasal application of PAF slightly delayed the mortality rate of the animals in this experiment and its peritoneal co‐administration with amphotericin B even prolonged the survival suggesting antifungal synergism between the two compounds.^64^ A murine vulvovaginitis model proved that NFAP2 significantly decreases the cell number of fluconazole‐resistant *C. albican*s during the infection; furthermore its combination with fluconazole enhances the activity.^60^ These results promise a safe *in vivo* administration of crAFPs as mono‐ or polytherapeutic agents in treatment of fungal infection. However, the cation‐sensitivity discussed above combined with poor bioavailability could diminish their potential application as systemic agents in an anti‐*Candida* therapy. *In silico* predictions show a possible high binding affinity of crAFPs to human serum albumin based on the calculated low binding free energy and dissociation constant (Table 2).^65^ Keeping these limitations in mind, the topical application of crAFPs to treat dermal and mucosal infections could be more promising.

## 6 *De Novo* Designed Synthetic Peptide Derivatives of crAFPs

### 6.1 Structure‐Activity Determinants of crAFPs for Anti‐*Candida* Activity

Functional mapping combined with antifungal activity testing of synthetic peptide fragments derived from crAFPs provides a feasible basis to study their structure‐activity determinants.^50^ These antifungal active peptide motifs can serve as potential new antifungal compounds or as templates for rational peptide design to improve the features of the protein to meet the requirements for safely applicable and effective antifungal therapy.^26^,^50^ The crAFPs from Eurotiomycetes that have already been isolated and characterized as well as those which can be predicted by genome mining contain an evolutionary highly conserved consensus sequence ([GXC]‐[X_3‐9_]‐[C]) that can also be found in small and Cys‐stabilized antimicrobial proteins from other organisms.^26^,^66^ This motif, the so‐called γ‐core, localizes in the flexible loop regions at the N‐ or C‐terminus (Figure 3), or in the center of crAFPs in forward (dextromeric isoform) or reverse direction (levomeric isoform) (Table 1).^26^ The impact of the γ‐core motif on the anti‐*Candida* activity has been investigated recently in PAF. A synthetic peptide spanning this PAF motif located in loop 1 exhibited antifungal activity *per se*.^26^ This antifungal efficacy strongly depends on the amino acid composition. The increase of the peptide net charge (at pH 7) and of its hydrophilicity by respective amino acid substitutions dramatically improved the antifungal efficacy of the synthetic γ‐core peptide.^26^ Similarly, the anti‐*Candida* activity of PAF could be significantly increased when the same amino acids as in the synthetic peptides were exchanged in the γ‐core of the full‐length protein.^26^ In contrast, the C‐terminally located γ‐core of NFAP2 seems to play no role in anti‐*Candida* activity, most probably, because this motif has an almost neutral charge and contains numerous hydrophobic amino acids.^50^ Instead, the NFAP2 γ‐core presumably supports correct protein folding.^50^ Interestingly, a synthetic positively charged and hydrophilic peptide deduced from loop 1 of NFAP2 was found functionally active.^50^

### 6.2 *In Vitro* Antifungal Effect of crAFP Peptide Derivatives

Synthetic peptides with antifungal activity spanning the PAF γ‐core and the N‐terminal loop 1 of NFAP2 are unstructured, and less active than the full‐length proteins (Table 3 and Table 4).

**Table 3:** Amino acid sequence and *in silico* predicted physicochemical properties of synthetic crAFP‐derived anti‐*Candida* peptides.

Number of amino acids	Molecular weight (kDa)^34^	Number of Cys	Number of Lys/Arg/His	Theoretical isoelectric point^34^	Estimated charge at pH 7^35^	GRAVY^34^
NFAP2 mid‐N terminal region (Fr‐4): NNC(−SH)KHKKGSGC(−SH)^50^						
11	1.2	2	3/0/1	9.39	+3.1	−1.682
PAF γ‐core (Pγ): KYTGKC(−SH)TKSKNEC(−SH)K^26^						
14	1.6	2	5/0/0	9.51	+3.8	−1.814
PAF γ‐core variant (Pγ^var^): KYTGKC(−SH)YKKKNEC(−SH)K^26^						
14	1.7	2	6/0/0	9.63	+4.8	−2.079
Rational designed PAF γ‐core (Pγ°^pt^): KYTGKC(−SH)KTKKNKC(−SH)K^26^						
14	1.7	2	7/0/0	10.04	+6.8	−2.064

GRAVY: grand average of hydropathy value.

**Table 4:** Minimal inhibitory concentrations (MIC) of synthetic peptides spanning the γ‐core motif of NFAP2 and PAF (see Table 3) on *C. albicans* and NACs.

Species	MIC (μM)			
	NFAP2^50^	PAF^[26]*^		
	Fr‐4	Pγ	Pγ^var^	Pγ°^pt^
*C. albicans*	43	10	2.5	1.3
*C. glabrata*	n.d.	>20	n.d.	>20
*C. krusei*	43	20	n.d.	2.5
*C. parapsilosis*	43	10	n.d.	1.3

*The MIC of the synthetic PAF‐derived γ‐core peptides on NACs was determined as described.^26^ n.d.: not determined.

This lower efficacy could be the consequence of their non‐specific antifungal mode of action against yeasts that differs from that of the full‐length protein (*e. g*. immediate plasma membrane disruption) or because other protein motifs may contribute to full antifungal activity.^26^,^50^ The anti‐*Candida* activity of these peptides does not depend on the primary structure, because scrambled peptide variants thereof show the same efficacy as the native peptides.^26^,^50^ This observation allows the conclusion that the overall physicochemical properties (such as the net charge and the hydrophilicity in Table 3) determine the anti‐*Candida* efficacy, and the peptides do not have amino acid sequence‐specific binding targets in the yeast cell. Rational design of the PAF γ‐core peptide supports this claim (Table 3). Specific amino acid substitutions that elevate the positive net charge and reduce the hydrophobicity render the peptide variant similarly effective against *C. albicans* and NACs than the full‐length protein variant (Table 4). These rational *de novo* designed PAF γ‐core peptide variants do not show hemolytic activity and do not affect the viability of primary human skin cells, which promises their potential therapeutic application.^26^

## 7 Summary and Outlook

The outlined features render the crAFPs and peptide derivatives promising candidates for potential clinical anti‐*Candida* therapy. However, the poor pharmacokinetic properties, and the reduced antifungal efficacy in high ionic strength environment hampers their applicability. Rational design and appropriate formulations might overcome these limitations. Considering the increasing number of antifungal drug‐resistant *Candida* strains and the fact that *Candida* species mainly affect the skin or mucosa posing a risk to become fatal invasive infections in immunocompromised patients the administration of crAFPs or peptide derivatives could be more promising than treatment with a conventional drug.^67^ The crAFPs could also be considered as substances in combination drug products to facilitate the antifungal effect of already applied compounds.^68^ Different formulations of crAFPs and their peptide derivatives, such as liposome or nanocarrier encapsulation, could improve the bioavailability and enhance the pharmacokinetic properties as it has been proven already for several antibacterial peptides.^69^ In addition to pharmacokinetic improvements, further intensive *in vivo* investigations focusing on the therapeutic application are essential to facilitate the introduction of crAFPs in clinical therapy.

## Biographical Information

*Annie Yap finished her B.Sc. degree in Applied Health Science, specializing in Clinical Diagnosis from Manipal Academy of Higher Education (MAHE), Manipal (India) in 2009. She is currently pursuing her M.Sc. in Microbiology at the Leopold‐Franzens University of Innsbruck (Austria). Since January 2018 she is working on her master thesis, with antimicrobial proteins and peptides from filamentous fungi as the main area of research, under the supervision of Florentine Marx, Division of Molecular Biology, Biocenter, Medical University of Innsbruck*.

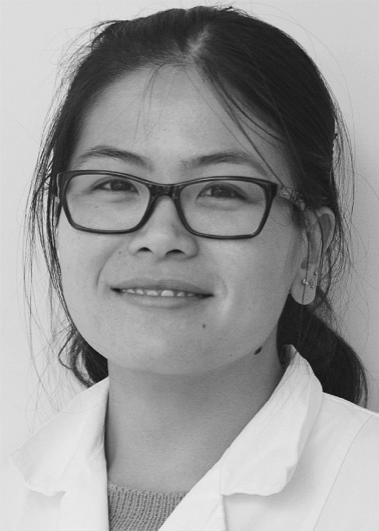

## Biographical Information

*László Galgóczy received his PhD in Microbiology from the University of Szeged (Hungary) in 2008. During the first period of his scientific career (2008‐2014), he had continuous grant support by national funds to start investigations on antifungal proteins at the same institute. In 2014 he obtained the international Lise Meitner Fellowship from the Austrian Science Fund to achieve his research program on structural investigation of antifungal proteins in Florentine Marx's laboratory between 2014 and 2016. After return to Hungary he joined to the Biological Research Center of Hungarian Academy of Sciences and to the University of Szeged; and he has been continuing the established collaboration with Florentine Marx to design novel antifungal proteins and peptides with improved efficacy*.

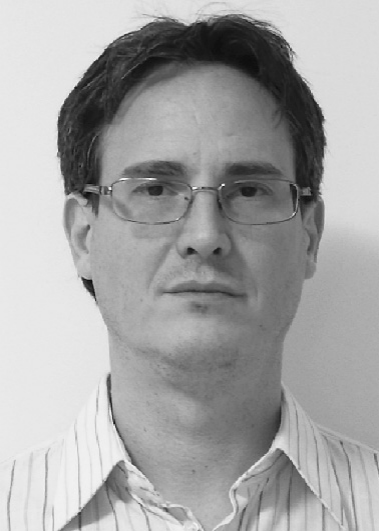

## Biographical Information

*Florentine Marx received her PhD in Microbiology from the Leopold‐Franzens University of Innsbruck (Austria). After post‐doctoral studies at the Institute of Biomedical Aging Research/Austrian Academy of Sciences in Innsbruck and the Institute of Virology and Environmental Microbiology/National Environmental Research Council in Oxford (UK), she became assistant professor in 1999 and habilitated in Microbiology at the Medical University of Innsbruck (MUI). Since 2006 she is associate professor and leads the “Applied Mycology Group” at the Biocenter, Division of Molecular Biology (MUI). The focus of her research is on the characterization of the mode of action and the structure‐function relation of small, cysteine‐rich, cationic antimicrobial proteins (AMPs) from filamentous Ascomycota*.